# Some Affections of the Upper Air-Passages

**Published:** 1880-01

**Authors:** Samuel Johnston

**Affiliations:** Baltimore


### SOME AFFECTIONS OF THE UPPER AIR-PASSAGES.

By SAMUEL JOHNSTON, M.D. Baltimore.

The laryngoscope has become a *sine qua non* in practical medicine, and the profession are daily availing of the specialist in this department of medical science. It occurs to me, therefore, that it will not be superfluous, considering the prominent place which this instrument holds as a means of diagnosis, and aid to treatment in affections of the upper air-passages, to report a few cases of laryngeal and nasal disease—I have thus chosen such cases as I think will be especially interesting to the general practitioner.

Case 1. *Papillomatous Growth on the Right Vocal Cord.* Mr.

J., aged 53, a builder by occupation, consulted me some time since for dysphonia of nine months duration.

His business required constant straining of his voice, and frequent exposure to the vicissitudes of the weather. For two years past his voice has been gradually failing him; but not until the last few months has he become almost completely aphonic. He has a croupous cough, and a "constant feeling of obstruction in his throat." When I first saw him it was with difficulty that he could make himself understood. Talking was painful, but he had no dyspnœa or difficulty in swallowing. His general health was excellent.

The laryngoscopic mirror revealed a papillomatous growth the size of a small raspberry, pearly white in appearance, situated upon the right vocal-cord, occupying its free edge, and to a certain extent its under surface.

On inspiration, the tumor floated freely in the trachea, being pushed downwards by the thickened ventricular-band; but on attempted vocalization, the growth projected across the glottis. The whole of the laryngeal mucous membrane was in a state of chronic catarrh. After educating the throat by the daily introduction of the mirror and laryngeal sound, I was enabled in the course of a few days, with Mackenzie's forceps, to remove one-half of the growth, and at a subsequent sitting two days later I succeeded, without difficulty, in removing the whole of the remaining portion.

A slight amount of laryngeal hyperæmia followed the operation; but this was soon reduced by the vapor benzoin, and mild astringent sprays. A month after the removal of the growth the voice was fast regaining its former clearness, and would have been completely restored but for the thickened ventricular band which prevented the free vibration of the vocal cord on the corresponding side.

CASE 2. *Tertiary Syphilitic Throat, with Stenosis of the Larynx.* E. B., female, 43 years of age, was sent to me by Dr. Riggen Buckler, in February, 1877.

I could get no history of a primary syphilitic lesion; but for several years she had suffered at times from sore-throat, and a husky voice. When she consulted me her voice was almost extinct, respiration was much embarrassed, attacks of laryngeal spasm increased her distress, and she swallowed with great pain. Upon laryngoscopic examination, the soft palate was seen to be firmly united to the posterior pharyngeal wall, a small opening alone being left at the site of the destroyed uvula. The cicatricial tissue was firm, tense, and glazed in many places. The laryngeal mucous membrane was deeply congested, and the neighboring parts much swollen; the glottis was nearly closed and covered with tenacious mucous; the vocal cords were completely destroyed, and the ventricular bands showed evidence of former ulceration.

A large ulcer, covered with a grayish colored deposit, was situated at the base of the right arytenoid cartilage; the epiglottis was twisted upon itself, but had escaped the destructive process.

Although tracheotomy was imminent, I decided to try first the iodide of potassium. Twenty-five grains of the salt, with three grains of the carbonate of ammonia, in a decoction of bark, was therefore ordered every four hours. She was also directed to inhale vapor benzoin every hour, and to take plenty of liquid nourishment. The ulcer was touched with a solution of the sulphate of copper, and the neck over the thyroid cartilage and neighboring parts was painted with the tincture of iodine.

By the fourth day the distressing symptoms had greatly improved. The iodide of potassium was now reduced to ten grains four times daily, and the other medication was continued.

At the end of the second week the tumefaction about the larynx had quite subsided, and the ulcer at the base of the arytenoid cartilage was healed. She could swallow without inconvenience, and was in all respects very much better. Anti-syphilitic treatment was continued for some months, when she was quite well. The voice was good—though gruff in tone; the larynx was healthy, but showed clearly the ravages of the disease.

A recent writer upon tertiary affections of the larynx, says,—"Tertiary affections of the throat are extremely common, and, when not treated they occasion loss of voice and difficulty in deglutition; and there are few cases where the practitioner is more liable to allow grave and irreparable evil to ensue if he mistakes the disease, or does not know how to apply a timely remedy to it."

CASE 3. *Acute Laryngitis Caused by the Inhalation of Coal Dust.* L. H., 16 years of age, a robust servant girl, was taken ill in February of this year.

Having become quite suddenly hoarse, with accompanying febrile symptoms, the family physician was sent for. The age of the girl, and the fact that she had never menstruated, led the doctor, in the first instance, to regard the case as one of aphonia, partially of a hysterical and partially of a catarrhal nature.

The symptoms, however, not yielding to the usual remedies; but, on the contrary, gradually increasing laryngeal obstruction becoming evident, I was requested to see the case.

When I saw the girl her voice was reduced to a whisper, and her breathing labored, especially on inspiration; the prolabiæ were lived, and the whole countenance showed evidence of the insufficient oxygenation of the blood. Add to these objective symptoms a croupous cough, a frequent pulse, a skin freely perspiring, and a dry mouth and tongue.

Upon interrogating the patient, I found that the day previous to the beginning of her attack, she was in a cellar sifting coal ashes, and was for some hours exposed to the dense volumes of dust arising from this process. Examination with the laryngeal mirror showed a pharynx normal in all respects.

The epigolttis, ventricular bands, vocal cords and surrounding parts in a state hyperæmia, much swollen, and covered with a thin layer of tenacious mucus.

Below the vocal cords was seen a grayish colored mass, resembling false membrane, firmly adhering to the under surface of the vocal cords, and tracheal wall, and, by its quantity and particular situation greatly obstructing the entrance to that tube. An oval opening about the size of a small quill, with irregular edges was seen in the centre of this mass, through which alone respiration was carried on.

Her condition was critical, and I feared tracheotomy, not so much from the obstruction in the trachea, *per se* as from the attacks of laryngeal spasm which frequently attend inflammatory conditions of these organs.

She was directed to inhale an atomized solution of the chloride of sodium, with a few drops of carbolic acid added.

After inhaling some drachms of this solution which had the effect of softening the deposit in the trachea, she was urged to cough violently. The mass was in this simple manner dislodged from its attachments and expelled. The dyspnœa was at once relieved, and the voice restored. The patient was ordered in bed, and in addition to ice to suck, a mixture of tr. aconite rad; vin. antimonii; in liqr. ammon. acetat. was prescribed. From this time her condition steadily improved. The deposit was not reproduced, and the laryngeal and tracheal hyperæmia which remained, soon yielded to astringent sprays, with the complete restoration of the normal function of the larynx.

Under the microscope, the mass expelled from the trachea was found to have some resemblances to a pseudo-membrane, and loaded with minute particles of coal dust.

CASE 4. *Retro-pharyngeal Abscess.* I was consulted by Mrs. M, concerning her baby twelve months old; whom she stated had great difficulty both in respiration and deglutition.

The child was strong and well until two weeks ago, when after taking a severe cold she noticed that his neck was stiff, that he nursed badly, and snored much while sleeping. When I saw the child, his breathing was very much embarrassed. Fluids taken into the mouth regurgitated through the nose; and he had lost much flesh from inability to take his accustomed quantity of nourishment.

There was no evidence of any disease of the vertebræ. On looking into the child's mouth, I observed a bulging forwards of the posterior pharyngeal wall, but not sufficient to account for the severity of the symptoms. Upon digital examination, however, I felt quite a large swelling, which imparted to the finger a distinct sense of fluctuation extending upwards behind the tonsil and downwards as far as the base of the epiglottis, which was pushed to one side by it. There was also a swelling externally at the angle of the jaw in front of the sterno-cleido-mastoid muscle on the corresponding side. Remembering the danger of opening an abscess in this situation, especially in so young a child, in the usual way, it occurred to me to perform the operation in the following manner:

Taking an ordinary tonsil bistoury guarded to within the one-eighth of an inch of the point, and steadying the swelling between the fore and middle finger of my left hand, I punctured the abscess as high up as possible, and immediately inverted the child; I then passed my fore finger into his mouth and made compression on the abscess from below upwards, the contents of the abscess escaping from the child's mouth into a vessel held by an assistant. The quantity of pus evacuated was about one ounce. The child now breathed with ease, and swallowed without difficulty.

Several weeks later there had been no re-accumulation of the pus, and the health of the child was completely restored.

CASE 5. *Abscess of the Nasal Septum.* Miss B., aged 25, was in good health, she states, until February, 1878, when one morning she noticed that her nose was inflamed, and she had a chill, followed by high fever. The family physician was consulted, and her affection diagnosticated erysipilas; the usual remedies in the treatment of this disease being prescribed. With time, the whole phase of the affection was changed, and I was asked to see the case five days after the beginning of her illness.

The physiognomy of the patient was peculiar. Her nose, face, and eye-lids were greatly swollen, deeply reddened and pitted upon pressure.

Protruding about the eighth of an inch from each nostril was a polypoid mass—fluctuating upon palpitation, attended by a muco-purulent discharge. The patient complained of a throbbing pain in the parts and dull frontal head-ache; the conjunctivæ were congested, and there was a constant overflow of tears in consequence of the temporary closure of the nasal ducts; hearing was also impaired, and the voice had a nasal twang. Examination with the mirror, showed the nasal mucous membrane deeply congested and much swollen.

Protruding about the eighth of an inch from each nasal orifice was a mass so extensive in size, as to completely occlude the nasal passages. The soft palate was inflamed, as was also the pharynx.

Rhinoscopic examination revealed tumefaction of the mucous surfaces in the post nasal space, and (the case being an unusually favorable one for rhinoscopy) the abscess was seen arising from the nasal septum, at the junction of the osseous and cartilagenous portions, projecting forwards. The abscess was opened, a free discharge of pus and blood giving, at once, great relief to the intense pain which the patient was suffering. Warm anodyne applications were made externally, and a solution of borax and carbolic acid, in tepid water, was injected through the nostrils every few hours. Quinine and iron were ordered, with beef tea and milk.

The patient made a satisfactory recovery from the acute symptoms. But necrosis of the nasal septum followed, the cause of which it was subsequently ascertained was due to specific disease.—*Maryland Medical Journal.*

